# Comparative larval ultramorphology of some myrmecophilous Aleocharinae (Coleoptera, Staphylinidae), with a first description of the larvae of *Amidobiatalpa* (Heer O, 1841) and *Oxypodahaemorrhoa* (Mannerheim C.G., 1830), associated with the *Formicarufa* species group

**DOI:** 10.3897/zookeys.808.29818

**Published:** 2018-12-18

**Authors:** Bernard Staniec, Mirosław agaja, Ewa Pietrykowska-Tudruj, Grzegorz K. Wagner

**Affiliations:** 1 Department of Zoology, Maria-Curie Skłodowska University, Akademicka 19, 20-033 Lublin, Poland Maria-Curie Skłodowska University Lublin Poland; 2 Isobolographic Analysis Laboratory, Institute of Rural Health, Jaczewskiego 2, 20-090 Lublin, Poland Isobolographic Analysis Laboratory, Institute of Rural Health Lublin Poland

**Keywords:** Aleocharines, Red wood ants, Coleoptera, developmental stages, ecological preferences, external structure, late larval instars, larva, morphology, myrmecophile, rove beetles, symbionts

## Abstract

The paper describes the external structures of the late larval stages of two Palearctic myrmecophilous staphylinids: *Amidobiatalpa* and *Oxypodahaemorrhoa* associated with the *Formicarufa* species group. This is the first-ever description of the larva of *Amidobia*, and the only complete, detailed account of the morphology of this developmental stage in the genus *Oxypoda* currently available. For the first time in these two genera, 13 and 10 larval diagnostic features, respectively, are proposed. Morphological differences have been established between known and the newly described larvae of five species (genera) of myrmecophilous and one non-myrmecophilous Aleocharinae, belonging to three tribes. *Amidobiatalpa* and *O.haemorrhoa* are probably typical, tiny predators, like most other Aleocharinae, including non-myrmecophilous ones. Being very small and highly mobile, they are ignored by worker ants. Not surprisingly, no particular larval morphological modifications were found to enable them to survive among ants. Such features have, however, evolved in the larvae of larger aleocharines, that is, those that are perceived by ants and are wholly integrated with their hosts in the ant nest (e.g. *Lomechusa*). This comparative analysis of the functional morphology of the larvae of known myrmecophilous Aleocharinae is a springboard to further such studies of these interesting insects.

## Introduction

Red wood ants from the *Formicarufa* Linnaeus, 1761 species group are regarded as key insect species in European woodlands because of their vast numbers and the invaluable biocoenotic contribution they make to the ecosystems they inhabit. Being polyphagous predators, they have a major, multidimensional effect on the invertebrate fauna in that they limit the numbers of many harmful woodland phytophages. On the other hand, the presence of ants has a very positive effect on a range of tiny woodland creatures, such as aphids, which ants protect and defend, obtaining honeydew in return (Szujecki 1998, Laakso and Setälä 2000). Moreover, these ants build complex nests with an extensive inner space, which provides a distinctive microclimate with stable levels of temperature and humidity, as well as constant and diverse food resources. It is therefore an optimal but at the same time highly specific microhabitat for a great number of myrmecophilous invertebrates, predominantly insects (Hölldobler and Wilson 1990, Päivinen et al. 2002, Staniec and Zagaja 2008, Parmentier et al. 2014, Parker 2016).

Among the insects associated with ants, beetles (Coleoptera) are the richest and the most diverse in form. According to Hölldobler and Wilson (1990), there are many thousands of myrmecophilous coleopteran species in 35 families. By way of example, as many as 166 species of beetle have been found in the nests of *Formicarufa* (Päivinen 2002). By far the most numerous group of myrmecophilous beetles are from the family Staphylinidae (rove beetles), the majority of which, in turn, belong to the subfamily Aleocharinae (Wilson 1979, Kistner 1979, Kronauer and Pierce 2011, Parker 2016) represented, among others, by *Amidobiatalpa* Heer O, 1841 and *Oxypodahaemorrhoa* Mannerheim C.G., 1830.

The genus *Amidobia* Thomson C.G., 1858 (Athetini) contains eight Palearctic species, of which only *A.talpa* (Heer O., 1841) occurs in Europe (Löbl and Löbl 2015). This is a very small myrmecophile (body length ca 1.5 mm), inhabiting mainly the nests of *Formicarufa* and related species (Lohse 1974, Burakowski et al. 1981, Koch 1989). To date, this rove beetle has been reported from the nests of *Formicarufa*, *F.pratensis* Retzius, 1783, *F.aquilonia* Yarrow, 1955, *F.polyctena* Foerster, 1850, *F.lugubris* Zetterstedt, 1838, *F.truncorum* Fabricius, 1804, *F.execta* Nylander, 1846 and *Lasiusfuliginosus* Latreille, 1798 (Päivinen et al. 2002, 2003, Staniec and Zagaja 2008, Parmentier et al. 2014). Its distribution range lies in central Europe and Fennoscandia, extending beyond the Arctic Circle in the north, and reaching the Caucasus, Siberia and the north of the Korean peninsula in the east. It is probably present throughout Poland, but there are still no records of it from many regions (Burakowski et al. 1981, Löbl and Löbl 2015).

The genus *Oxypoda* Mannerheim, 1830, one of the most species rich aleocharine genera, has a worldwide distribution (Newton et al. 2000, Löbl and Löbl 2015). *Oxypodahaemorrhoa* (Mannerheim C.G., 1830) (Oxypodini) belongs to the subgenus *Bessopora* Thomson, 1859, which has 100 species in the Palearctic, 13 of which occur in Poland (Melke 2014, Löbl and Löbl 2015). This rove beetle is widely distributed from North Africa across continental Europe as far as Iceland and northern Fennoscandia; it has also been recorded in Siberia. In Poland, it probably inhabits all parts of the country except the higher mountain areas, although some regions currently lack records (Burakowski et al. 1981). This myrmecophile is one of the smaller European representatives of the genus (body length: 2.0–2.7 mm). It lives mainly in ant nests from the *Formicarufa* group: *F.rufa*, *F.pratensis*, *F.polyctena*, *F.aquilonia* and *F.lugubris*, but one also comes across it in the nests of *F.truncorum*, *F.execta, F. sanguinea* Latreille, 1798, *F.suecica* Adlerz, 1902, *F.nigricans* Emery, 1909 and *Lasiusfuliginosus* (Päivinen et al. 2002, 2003, Staniec and Zagaja 2008, Lapeva-Gjonova 2013, Parmentier et al. 2014). It has sometimes been found in the neighbourhood of anthills, under plant debris (Burakowski et al. 1981).

Myrmecophilous Aleocharinae, like other beetles associated with ants, are fascinating organisms for research because of their highly interesting morphological, ecological and behavioural adaptations to the distinctive conditions found in ant nests (Kolbe 1971, Hölldobler and Wilson 1990). In this context, however, very few data are available concerning the structure of their larval stages. Contemporary data on this subject relate to just three species: *Pellalaticollis* Märkel F., 1844, *Thiasophilaangulata* (Erichson W.F., 1837) and *Lomechusapubicollis* Brisout de Barneville Ch.N.F., 1860. The first-mentioned is associated primarily with *Lasiusfuliginosus*, the other two with ants from the *Formicarufa* group (Staniec et al. 2009, Zagaja et al. 2014, Staniec et al. 2017).

Presumably, the larval structure of these myrmecophiles, among other characteristics, which actively live and forage in the anthill throughout their development, should well reflect the extent and nature of their integration with their hosts. Therefore, detailed morphological data of the larval forms should prove useful for discovering the distinctive adaptations of myrmecophilous species to life in ant nests and also the relations between them and their hosts.

The links of numerous Staphylinidae with such a characteristic habitat like ant nests are reflected in various degrees of specialization. In the context of host-guest interactions, Wasmann (1894) classified arthropods inhabiting anthills into ectoparasites, endoparasites, trophobionts, synechtry, synoics and symphiles. Myrmecophilous aleocharines can be placed in these last three categories. *Lomechusapubicollis* is a representative of the symphilous Aleocharinae. Like all symphiles, this rove beetle is the most highly integrated with its host; evidence for this can be found *inter alia* in a number of adaptive morphological features in its larval stage, recently described by Staniec et al. (2017). In turn, both *Amidobiatalpa* and *Oxypodahaemorrhoa* are probably synoics, that is, myrmecophiles feeding on debris or other organisms inhabiting the anthill; being small and highly mobile, they are probably overlooked by their hosts. On the other hand, both these beetles will readily feed on ant larvae, thus exhibiting typical features of synechtry (Parmentier et al. 2015, own observations). The myrmecophilous *Thiasophilaangulata* (Erichson W.F., 1837) has a similar lifestyle (Zagaja et al. 2017), but the morphology of its larva is no different from that of the larvae of other, related but non-myrmecophilous aleocharine beetles (Zagaja et al. 2014).

It is apposite, therefore, to pose the following questions: 1) Does the myrmecophily of *A.talpa* and *O.haemorrhoa* have any effect on the external structure of their larvae? 2) Are their ecological preferences of no great importance in this respect, as in the case of *T.angulata*? 3) How does the extent of guest-host integration affect the morphology of aleocharine larvae so far examined?

The chief aim of this paper is therefore to describe in detail the morphology, including the chaetotaxies and external ultrastructure, of the larval stages of *Amidobiatalpa* and *Oxypodahaemorrhoa* and to compare them with the external larval structures of other, well-known myrmecophilous aleocharines.

## Materials and methods

### Material examined

Larval stages were obtained by rearing 34 adults of *Amidobiatalpa* and 9 adults of *Oxypodahaemorrhoa*. Specimens of both species were collected on May 5, 2017, at Lake Moszne (51°26'57.4"N, 23°07'34.0"E) and Lake Długie (51°27'04.0"N 23°09'39.9"E), situated in the Polesie National Park near Lublin (SE Poland). The insects were sifted from the nest material of *Formicapolyctena*. Live beetles of *A.talpa* and *O.haemorrhoa* were placed in transparent plastic containers (diameter 10 cm, height 4 cm) filled with nest substrate and observed in the laboratory from May 9 to June 24, and from May 11 to June 21, respectively, at room temperature (22–25 °C). Adults and larvae of various species of ants, including *F.rufa* and small springtails, were supplied as a source of adult food.

### Study techniques

Larvae of both species were killed in boiling water and preserved in ethanol (75%).

To prepare temporary microscope slides, some larvae were macerated in cold 10% KOH for two to three hours, immersed in lactic acid for subsequent preparation and mounting of antennae, mouthparts, sensory structures, chaetotaxy of the body, legs and urogomphi. They were then traced from photos taken with an Olympus DP72 or Olympus DP21 digital camera mounted on a binocular Olympus SZX16 or Olympus BX63 compound microscope (Figs 9, 13, 14, 17, 18, 22–46, 48, 49, 52, 53, 53a, 54, 54a, 55, 56). The final image adjustments were made using CorelDraw Graphics Suite 2018.

Habitus illustrations of larvae, structure of setae, chaetotaxy of head, functional position of mouthparts, structural details of antennae, microstructure, spiracles and various details of their external structure were recorded using SEM, type VEGA3 TESCAN (Figs 1–8, 10–12, 15, 16, 19–21, 42a, b, 43a, 44a, 47, 47a, 50, 51, 53b, 54b). For the SEM work, larval specimens taken from alcohol were briefly dried and placed directly in the SEM chamber for observation.

### Measurements and their abbreviations

Measurements of the larvae of both species, made using an Olympus BX63 compound microscope in cellSens Dimension v1.9 software, are given in millimetres, as explained in detail in Pietrykowska-Tudruj and Staniec (2012). Measurements (Table 1) were made on freshly killed specimens. The terms of morphological structures, chaetotaxy (selected aspects only) and their abbreviations generally follow Ashe and Watrous (1984) and Staniec et al. (2018), with modifications in some of the figures. The material examined for the measurements is listed in Table 1. The material examined for morphological descriptions includes four or five specimens of the late-instar larva of each species. The voucher specimens are deposited in the collections of the Department of Zoology, Maria Curie Skłodowska University, Lublin.

**Table 1.** Measurements of larval instars of *Amidobiatalpa* and *Oxypodahaemorrhoa*. Symbols and abbreviations: larval instars, A – average, N – number of specimens examined, M – measurement, R – range, SV – standard variation.

Species (larval instars/N)	M	R	A	SV
*Amidobiatalpa* (all larval instars/28)	Body length	1.00–2.55	1.99	0.45
	Thorax length	0.38–0.69	0.55	0.09
	Head width	0.16–0.23	0.20	0.02
	Prothorax length	0.15–0.30	0.21	0.40
	Prothorax width	0.18–0.27	0.23	0.03
*Oxypodahaemorrhoa* (all larval instars/17)	Body length	2.46–3.32	2.79	0.37
	Thorax length	0.40–0.80	0.68	0.09
	Head width	0.22–0.27	0.25	0.02
	Prothorax length	0.19–0.33	0.27	0.03
	Prothorax width	0.23–0.32	0.28	0.02

## Results

### Generic diagnosis of the late larval instar of *Amidobia* and *Oxypoda*

The combination of characteristics distinguishing mature larvae of *Amidobia* and *Oxypoda* from known larvae of other genera within the subfamily Aleocharinae are as follows (Paulian 1941, Pototskaya 1967, Topp 1975, Ashe 1981, Ashe and Watrous 1984, Ashe 1985, Ahn 1997, Jeon and Ahn 2009, Staniec et al. 2009, 2010, 2016, 2018, Zagaja et al. 2014, the present study): *Amidobia* – (1) body extremely slim; (2) head wider (1.1 ×) than pronotum; (3) sensory appendage of antennal segment II longer (1.1 ×) than antennal article III; (4) labrum rectangular; (5) anterior margin of labrum shallowly excised in the centre; (6) seta Ld2 of labium spiniform; (7) epipharynx with about 20 long cuticular processes and 2 pores centrally; (8) mandible stocky with 2 preapical teeth; (9) mala wide, distinctly dilated anteriorly; (10) adoral margin of mala with 10–12 teeth (4–5 small distally); (11) dorsal side of mala with about 25 cuticular processes; (12) ligula dome-like, as long as wide; (13) spiracle with unique perforation; *Oxypoda* – (1) head sides weakly rounded; (2) labrum semi-circular; (3) seta Ld2 of labium very short, verrucous; (4) epipharynx with about 100–150 short cuticular processes and 10 centrally; (5) mandible slender with 1 preapical tooth; (6) interior edge of apical and preapical tooth serrate; (7) adoral margin of mala with 11–15 teeth including 1 biggest proximally and a few small distally; (8) mala slender, narrowed anteriorly; (9) dorsal side of mala with about 15 cuticular processes; (10) ligula finger-like, almost twice as long as wide.

### Comparative morphological description of the late-instar larvae of *Amidobiatalpa* (*A.p.)* and *Oxypodahaemorrhoa* (*O.h.)*

**Figures 1–11.**
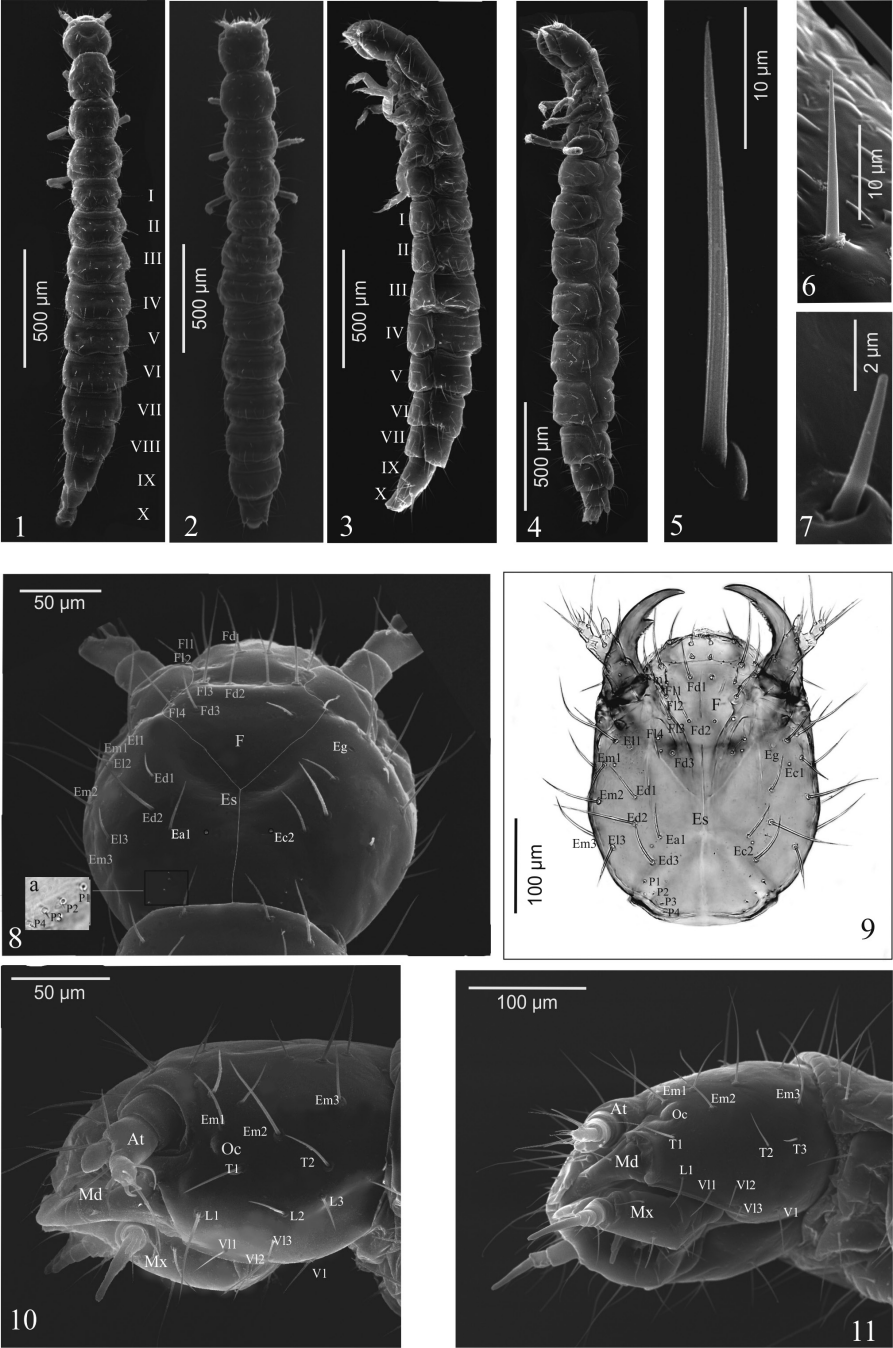
*A.talpa* (**1, 3, 5, 8, 8a, 10**), *O.haemorrhoa* (**2, 4, 6, 7, 9, 11**), mature larva. **1–4** habitus in dorsal (**1, 2**) and lateral (**3, 4**) aspect **5–7** abdominal setae **8–11** head in dorsal (**8, 9**) and lateral (**10, 11**) aspect. Abbreviations: I-X, abdominal segments; At, antenna; Ec, epicranial campaniform sensilla; Ed, epicranial dorsal setae; Eg, epicranial gland; El, epicranial lateral setae; Em, epicranial marginal setae; Es, epicranial suture; F, frons; Fd, frontal dorsal setae; Fl, frontal lateral setae; L, lateral setae; P, posterior setae; T, temporal setae; V, ventral setae; Vl, ventral lateral setae.

Body narrow, elongate, semi-cylindrical, segments IX and X distinctly narrower than the others; *A.p.* (Figs 1, 3) – head distinctly wider than prothorax and distinctly narrower than metathorax, abdomen gradually widening to segments V or VI, then tapering to terminal segment of body; *O.h.* (Figs 2, 4) – head distinctly narrower than pro- and metathorax, abdominal segments III-VII of more or less equal width. Colour: *A.p.* and *O.h.* – head poorly sclerotized, stemmata and mandible distinctly darker; thorax, legs and abdomen whitish, tergites somewhat darker, slightly and gradually darkening to terminal segments. Setae of different length, light brown, setose with longitudinal grooves (Figs 5–7).

**Figures 12–21.**
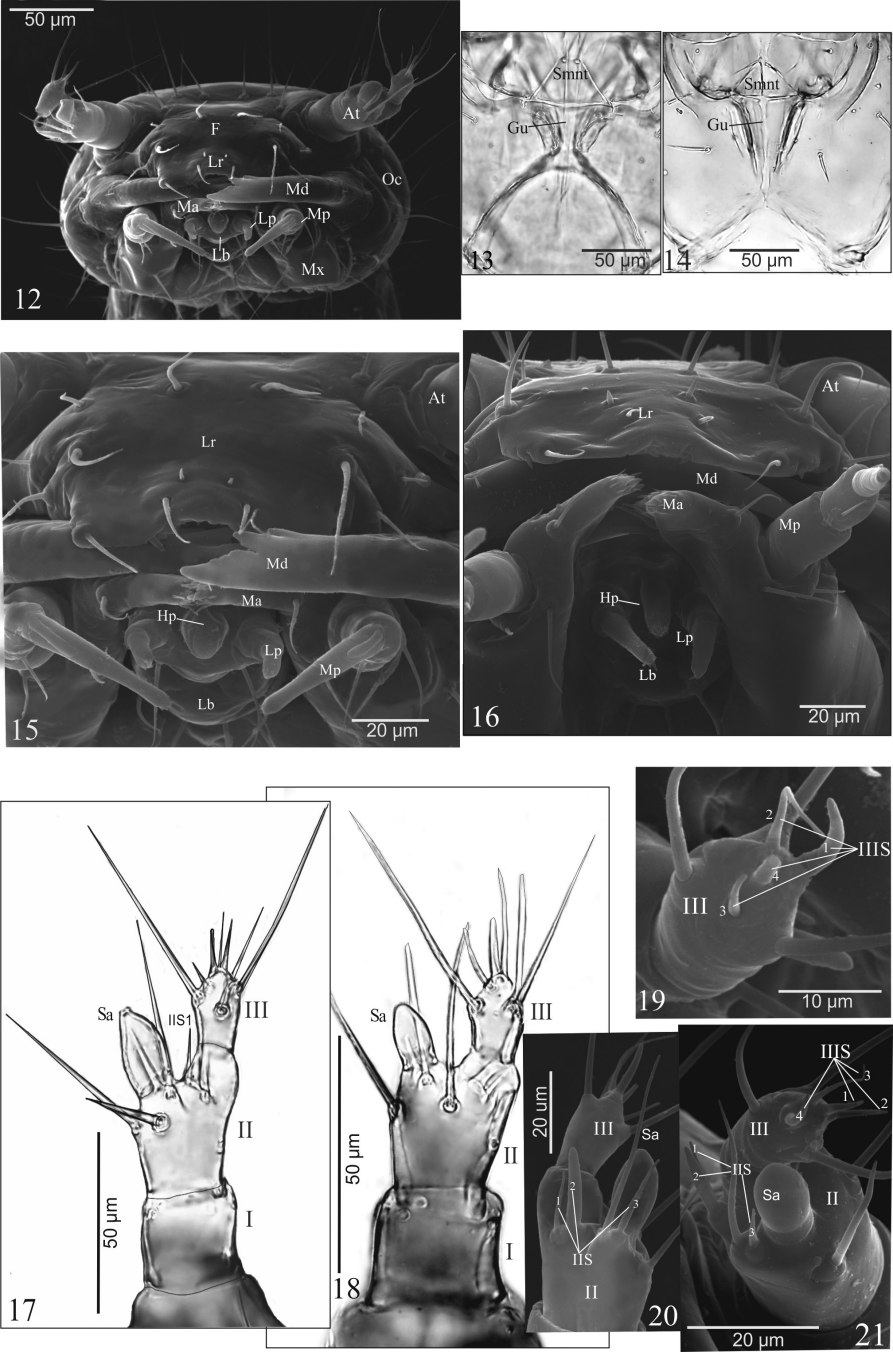
*A.talpa* (**12, 13, 15, 17, 19, 20**), *O.haemorrhoa* (**14, 16, 18, 21**), mature larva. **12** head in fronatl aspect **13, 14** gular region **15, 16** functional position of mouthparts in frontal aspect **17–21** right antenna in dorsal aspect (**17, 18**), entire article III in anterior (**19**) and in ventral (**20**) aspect, entire article II and III in anterior aspect (**21**). Abbreviations: I-III, antennal articles; IIS, IIIS, solenidia of antennal article II or III; At, antenna; F, frons; Gu, gula; Hp, hypopharynx; Lp, labial palp; Lr, labrum; Ma, mala; Md, mandible; Mx, maxilla; Pm, maxillary palp; Oc, ocellus; Sa, sensory appendage; Smnt, submentum.

**Head**. Shape: *A.p.* (Fig. 8) – distinctly (1.2 ×) wider than long, widest at level of setae El3, lateral margins distinctly rounded; *O.h.* (Fig. 9) – slightly (1.1 ×) longer than wide, widest at level of setae Em2, lateral margins moderately rounded. Chaetotaxy of dorsal side with 40 setae in *A.p.* – 14 frontal [2(Fd1–3, Fl1–4)], 18 epicranial [2(Ea1, Ed1–2, Ell-3, Em1–3)], 8 posterior micro setae (2P1–4), a pair of epicranial campaniform sensillae (Ec2) and epicranial glands (Eg) (Fig. 8); 42 in *O.h.* – 16 frontal [2(Fd1–3, Fm1, Fl1–4)], 18 epicranial [2(Ea1, Ed1–3, Ell, Fl3, Em1–3)], 8 posterior micro setae (P1–4), 2 pairs of epicranial campaniform sensillae (Ec1–2) and epicranial glands (Eg) (Fig. 9). Lateral margin with 10 setae [2(T1–2, L1–3)] in *A.p.* (Fig. 10); 8 setae [2(T1–3, L1)] in *O.h.* (Fig. 11). *A.p.* and *O.h.* – dorsal ecdysial lines (Es) bifurcate at about half the head length (Figs 8, 9); each side of head with one small, oval, weakly convex ocellus (Oc) (Figs 10, 11). Functional position of antennae (At), labrum (Lr), mandibles (Md), maxillae (Mx) with maxillary palps (Mp), mala (Ma), hypopharynx (Hp) and labium (Lb) with labial palps (Lp) as in Figs 12, 15 – *A.p.* and Fig. 16 – *O.h.* Gula (Gu) triangular: short, length to width ratio = 1 : 1.4 – *A.p.* (Fig. 13); elongate, length to width ratio = 1.5 : 1 – *O.h.* (Fig. 14).

**Antenna** (Figs 17–21): three-articled, length ratio of articles I–III: 1.5 : 1.0 : 2.0 – *A.p.* or 1.2 : 1.0 : 1.6 – *O.h.*. Article I: as long as wide – *A.p.* or 1.2 × wider than long – *O.h.* with 4 pores; article II 1.5 × as long as wide – *A.p.*, *O.h.*., with 3 macro setae, one acorn-shaped – *A.p.* or semi-spherical – *O.h.*., sensory appendage (Sa), 1.6 – *A.p.* or 1.9 – *O.h.* × as long as wide (Figs 17, 18), and 3 solenidia ventrally of different size (IIS1–3) – *A.p.*, *O.h.*. (Figs 20, 21); Sa 1.2 × longer – *A.p.* or 1.3 × shorter – *O.h.* than article III; article III 1.5 – *A.p.* or 1.6 – *O.h.* × as long as wide, with 3 macro setae and 4 solenidia apically (IIIS1–4) of different length and diameter (Figs 19, 21).

**Figures 22–37.**
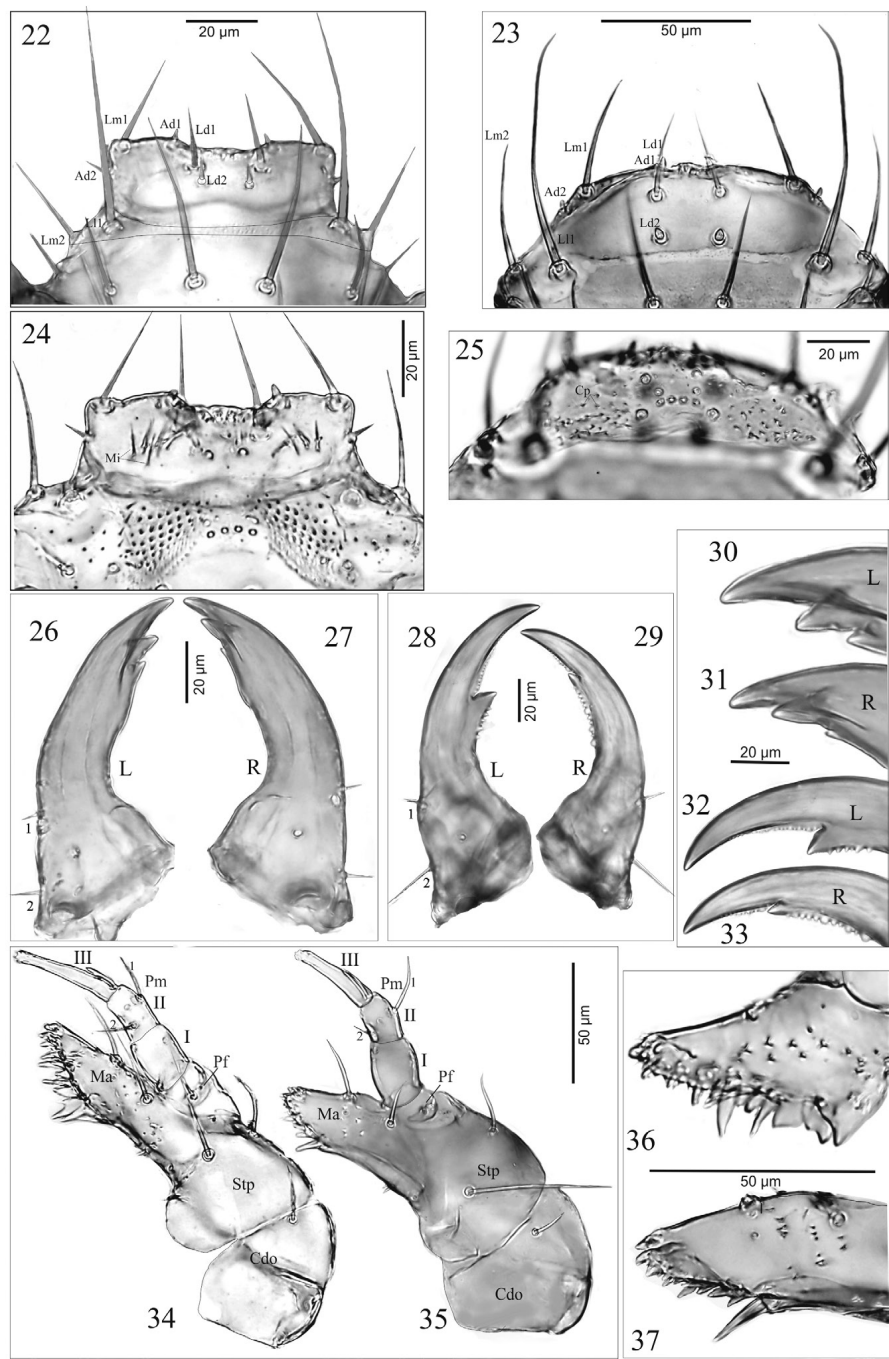
*A.talpa* (**22, 24, 26, 27, 30, 31, 34, 36**), *O.haemorrhoa* (**23, 25, 28, 29, 32, 33, 35, 37**), mature larva **22, 23** labrum **24, 25** epipharynx **26–29** left (L) and right (R) mandible, in dorsal aspect; **30–33** anterior region of left (L) and right (R) mandible, in dorsal aspect **34, 35** right maxilla in ventral aspect **36, 37** right mala in ventral aspect. Abbreviations: I-III, articles of maxillary palp; 1, 2, mandibular setae; Ad, additional setae; Cp, cuticular processes; Cdo, cardo; La, labral anterior setae; Ld, labral dorsal setae; Lm, labral marginal setae; Mi, microtrichia; Ma, mala; Pf, palpifer; Pm, maxillary palp; Stp, stipes.

**Labrum** (Figs 22, 23): posterior portion rectangular – *A.p.* (Fig. 22) or almost semi-circular – *O.h.* (Fig. 23) in outline, central region of anterior margin slightly excised – *A.p.* or rounded – *O.h.*; in *A.p.* length ratio of excised region and whole anterior margin 1:2.6; with 8 macro setae [2(Ld1, Lm1, Ll1, Lm2)], 2 micro setae (Ld2), spine-shaped centrally – *A.p.* or verrucous posteriorly – *O.h.*, and 4 additional short setae on anterior (Ad1) and lateral (Ad2) margin; separated from clypeal region by membranous area – *A.p.*, *O.h.*.

**Epipharynx** (adoral surface of labrum) membranous (Figs 24, 25), with two groups of a few microtrichia (Mi) each pointing towards anterior margin of labrum and a pair of pores centrally – *A.p.* (Fig. 24) or with sharp cuticular processes (Cp) directed towards central portion and 10 pores – *O.h.* (Fig. 25).

**Mandibles** (Figs 26–33): stocky, moderately bent, strongly widened basally – *A.p.* or slender, strongly bent, moderately widened basally – *O.h.*., with 2 micro setae (coded: 1 – upper, 2 – lower) on outer margin almost of equal length – *A.p.* (Figs 26, 27) or seta 2 distinctly longer than 1 – *O.h.*. (Figs 28, 29), and 2 pores; incisor lobe with one large subapical tooth and two such teeth of different size, the upper one larger than the lower one – *A.p.* (Figs 26, 27) or incisor lobe with 1 large and 1 smaller subapical tooth – *O.h.*. (Figs 28, 29); differences between left (L) and right (R) mandibles in *A.p.* and *O.h.*. as in Figs 30–33.

**Maxilla** (Mx) (Figs 34–37): consisting of triangular – *A.p.* (Fig. 34) or tetragonal – *O.h.* (Fig. 35) cardo (Cd) divided by sclerotized ridge into two unequal parts, wide stipes (Stp), obliquely truncate mala (Ma) distinctly widened – *A.p.* (Fig. 36) or slightly narrowed – *O.h.* (Fig. 37) at adoral margin, palpifer (Pf) and three-articled maxillary palp (Pm); cardo with one ventral seta; stipes with two setae; palpifer with one seta; mala fused with stipes, with two setae, one pore and approx. 25 – *A.p.* or about 15 – *O.h.* triangular cuticular processes ventrally; adoral margin of mala (functional positions in Figs 15, 16) with sparse group of a few micro teeth apically and ctenidium of 6 macro teeth, different sizes and shape proximally – *A.p.* (Fig. 36) or about 10 small teeth of equal length and 1 long tooth spinose proximally – *O.h.* (Fig. 37). Maxillary palp (Pm) (Figs 34, 35): length ratio of articles I–III: 1.1 : 1.0 : 2.0 – *A.p.* or 1.5 : 1.0 : 2 – *O.h.*.; article I slightly wider than second, 1.3 – *A.p.* or 1.4 –*O.h.*.× as long as wide with two pores; article II 1.5 – *A.p.* or 1.3 – *O.h.*× as long as wide with two setae (coded: 1, 2) equal in length – *A.p.* (Fig. 34) or unequal in length – *O.h.* (Fig. 35); article III narrower than I and II, tapering slightly to apex, 5.2 × – *A.p.* or 3.8 × – *O.h.* as long as wide, with one digitiform sensory appendage basally 0.4 × as long as article, one pore and a few tiny sensory apically.

**Figures 38–47.**
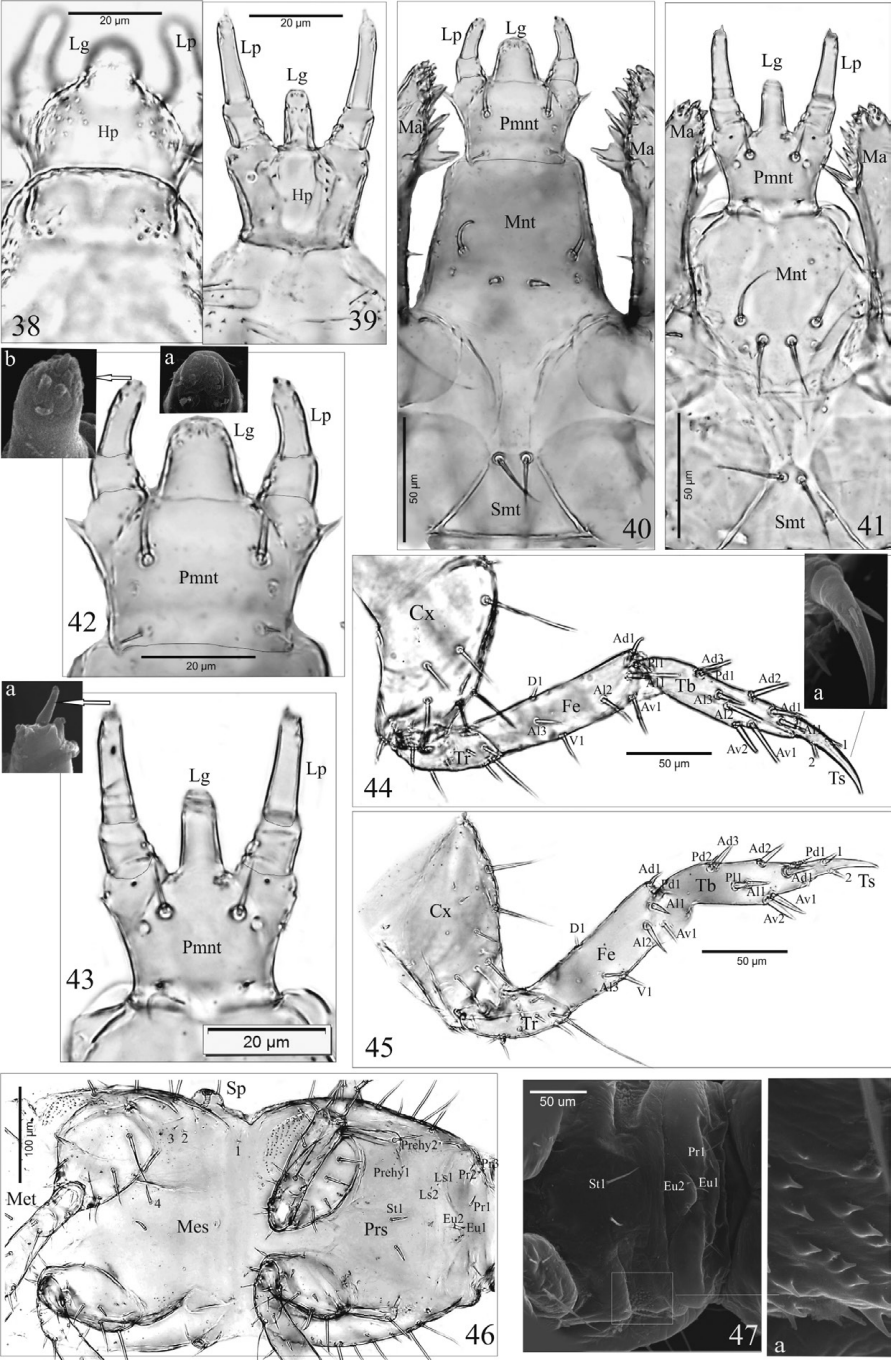
*A.talpa* (**38, 40, 42, 42a, b, 44, 44a**), *O.haemorrhoa* (**39, 41, 43, 43a, 45, 46, 47, 47a**), mature larva. **38, 39** hypopharynx **40, 41** labium **42, 43** prementum with labial palp, apex of ligula (**42a**) and labial palp (**42b, 43a**), **44, 45** legs and tarsungulus (**44a**), **46** pre- and mesosternum **47** anterior portion of presternum and microstructure (**47a**). Abbreviations: 1, 2, 3, 4, setae of mesosternum; 1, 2, setae of tarsungulus; Ad, anterodorsal setae; Al, anterolateral setae; Av, anteroventral setae; Cx, coxa; D, dorsal setae; Eu, eusternum; Fe, femur; Hp, hypopharynx; Lg, ligula; Lp, labial palp; Ls, laterosternum; Ma, mala; Mes, mesosternum; Mnt, mentum; Pd, posterodorsal setae; Pl, posterolateral; Pmnt, prementum; Pr, prosternum; Prehy, prehypopleuron; Prs, presternum; Smt, submentum; Sp, spiracle; St, sternellum; Tb, tibia; Tr, trochanter; Ts, tarsungulus; V, ventral setae.

**Hypopharynx** (Hp) (Figs 38, 39): membranous, surface (except central area) with approx. 25 – *A.p.* (Fig. 38) or a few tiny microtrichiae – *O.h.* (Fig. 39). Ligula (Lg): dome-like, gradually tapering to the top, as long as wide at base, separated from prementum by transverse line – *A.p.* (Figs 40, 42) or finger-like, tapering slightly to top, 1.9 × as long as wide, fused with prementum (Figs 41, 43); surface of apex with microsculpture resembling dermato-glyphics – *A.p.*, *O.h.* (Fig. 42a). Prementum (Pmnt) trapeziform, 1.3 × – *A.p.* or 1.1 × – *O.h.* as wide at base as long, with 4 setae (2 long, 2 short) and a pair of pores (Figs 42, 43). Labial palp (Pl) two-articled, length ratio of articles I and II: 1 : 2.4 – *A.p.* or 1 : 2.1 – *O.h.*, article I 1.4 × as wide as long – *A.p.* or as long as wide – *O.h.*, article II 2.9 × – *A.p.* or 3.1 × – *O.h.* as long as wide, with a few sensory appendages apically, almost equal in length – *A.p.* (Fig. 42b) or one sensory appendage distinctly longer than the others – *O.h.* (Fig. 43a).

**Thorax**. Foreleg (Figs 44, 45): consists of stocky coxa (Cx), short trochanter (Tr), elongate – *A.p.* (Fig. 44) or moderately elongate – *O.h.* (Fig. 45) femur (Fe), slim – *A.p.* or quite stocky – *O.h.*. tibia (Tb) and tarsungulus (Ts) slightly curving inwards (Fig. 44a); Cx, Tr, Fe, Tb and Ts: 1.8, 2,3, 3.6, 4.3 and 5.6 × – *A.p.* or 1.1, 2.1, 2.9, 3.0 and 3.5 × – *O.h.* as long as wide respectively; Fe, Tb and Ts with 8 (Ad1, Al1–3, Av1, D1, V1, Pl1 – *A.p.*, *O.h.*), 9 (Ad1–3, Al1–3, Av1–2, Pd1 – *A.p.* or Ad1–3, Al1, Av1–2, Pd1–2, Pl1 – *O.h.*) and 2 (1, 2) setae respectively. Length ratio of Fe, Tb and Ts: 1.8 : 1.7 : 1.0 – *A.p.* or 2.4 : 2.0 : 1.0 – *O.h.*.

**Figures 48–56.**
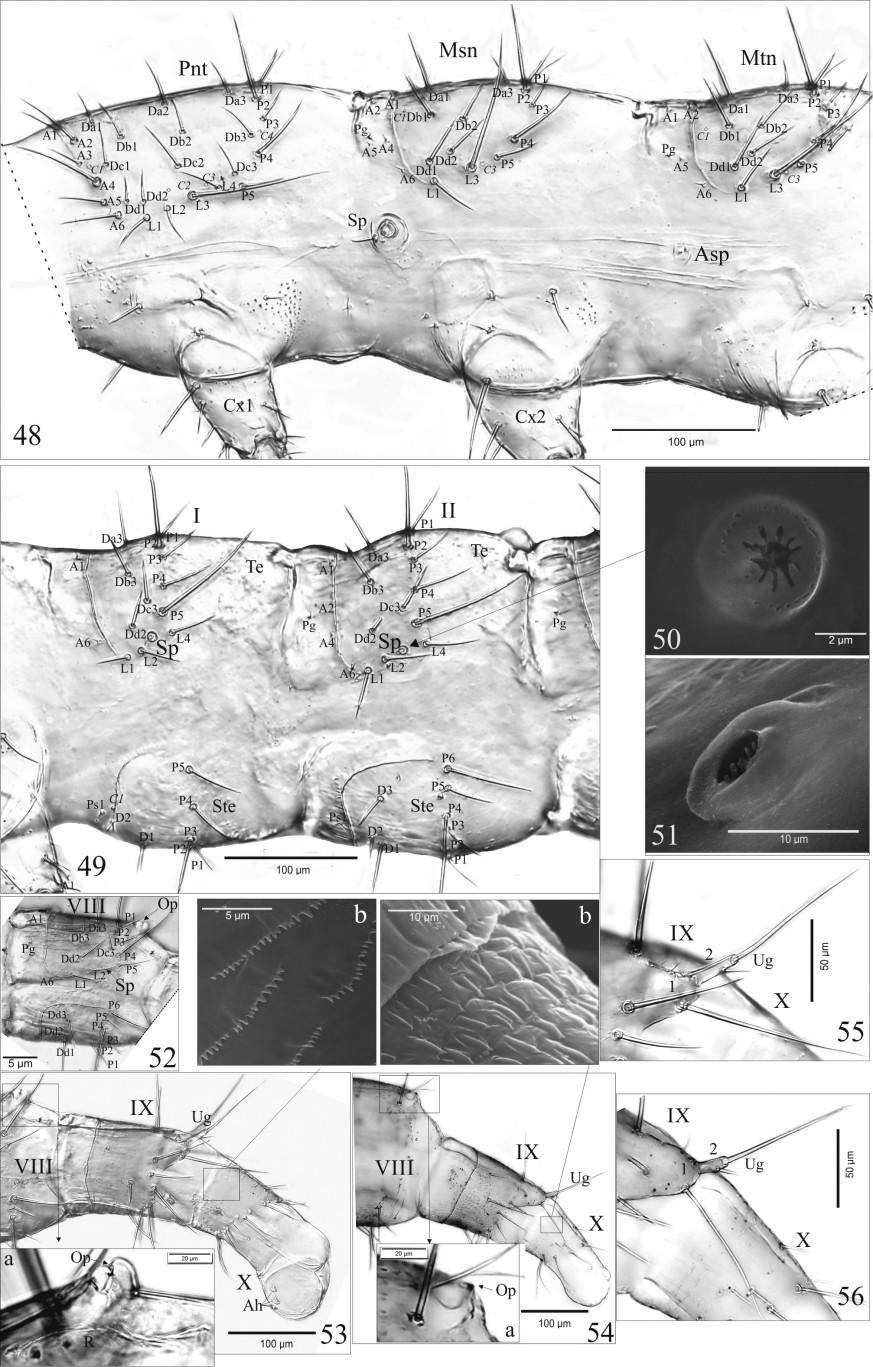
*A.talpa* (**48, 49, 50, 52, 53, 53a, b, 55**), *O.haemorrhoa* (**51, 54, 54a, b, 56**), mature larva. Thoracal (**48**) and abdominal segment I–II (**49**), VIII (**52**), VIII–X (**53, 54**) and urogomphus (**55, 56**) in lateral aspect. Abbreviations: 1, 2, article of urogomphi; VII – X, abdominal segments; A, anterior setae; Ah, anal hooks; Asp, atrophied spiracles; C, campaniform sensilla; Cx, coxa; D, Da-c, discal setae; L, lateral setae; Msn, mesonotum; Mtn, metanotum; Op, opening of gland reservoir; P, posterior setae; Pg, pretergal gland; Pnt, pronotum; Ps, presternal sensilla; R, gland reservoir; Sp, spiracle; Ste, sternite; Te, tergite; Ug, urogomphus.

Pro- (Prs), meso- (Mes) and metasternum (Met) membranous (Figs 46, 47): Prs with 20 setae [2(Eu1–2, Ls1–2, Pr1–3, Prehy1–2, St1)] and microstructure on sides (Fig. 47a), Mes and Met each with 8 setae (coded: 1–4). Prothorax (Pnt) 1.4–1.5 × as long as mesothorax (Msn), mesothorax and metathorax (Mtn) almost equal in length (Fig. 48): Pnt with 50 setae [2(A1–6, Da1–3, Db1–3, Dc1–3, Dd1–2, L1–4, P1–4)] and 8 pores (2[C1–4]); Msn with 36 setae [2(A1–2, A4–6, Da1, Da3, Db1–2, Dd1–2, L1, L3, P1–5)], 4 pores and 1 pair of paratergal glands (Pg), [(2C1, C3)]; chaetotaxy of metanotum identical with that of mesonotum; lateral area between pro- and mesothorax with a pair of large, functional spiracles (Sp), and between meso- and metathorax with a pair of atrophied spiracles (Asp) and one micro seta (Fig. 48).

**Abdomen**. Chaetotaxy of tergites: I with 28 setae [2(A1, A6, Da3, Db3, Dc3, Dd2, L1–2, L4, P1–5)], II-VII with 32 setae [2(A1, A2, A4, A6, Da3, Db3, Dc3, Dd2, L1–2, L4, P1–5)] and 1 pair of paratergal glands (Pg) (Fig. 49); VIII with 30 setae [2(A1, A6, Da2–3, Db2, Dc2–3, L1, L3–4, P1–5)], 2 pores (C5) and a pair of glands (Pg) (Fig. 52). Tergal gland reservoir (R) clearly developed with split opening (Op) at the posterior margin of abdominal tergite VIII; Op with unique structure for *A.p.* (Figs 53, 53a) and for *O.h.* (Figs 54, 54a). Chaetotaxy of sternites: I (Fig. 49) with 16 setae (2[D1–2, Ps1, P1–5]); II–VIII (Figs 49, 52) with 20 setae (2[D1–3, Ps1, P1–6]). Abdominal tergites I-VIII each with a pair of functional spiracles laterally (Sp) (Figs 49, 52) of unique structure for *A.p.* (Fig. 50) and for *O.h.* (Fig. 51). Segments IX and X distinctly narrower than the others with tergites and sternites fused in uniform ring (Fig. 53); segment IX with 24 setae (6 micro) (Fig. 53). Segment X with 14 setae and 4 anal hooks terminally (Ah) (Fig. 53) and unique microstructure for *A.p.* (Fig. 53b) and for *O.h.* (Fig. 54b). Urogomphi (Ug) of segment IX (Figs 53–56): two-articled, article I fused to tergum IX; article I (coded: 1) wide and turgid with 4 setae (3 macro); article II (coded: 2) slender, finger-shaped, moderately elongate and tapering apically, 2.8 × as long as wide at the base – *A.p.* (Figs 53, 55) or truncated, with sides almost parallel, 2.5 × as long as wide at the base – *O.h.* (Figs 54, 56), with 1 short seta subapically, 1 macro seta apically and a pore basally; length ratio of Ug and apical seta: 1 : 1.4 – *A.p.* or 1 : 1.3 – *O.h.*; length ratio of urogomphus (without apical seta) and segment X (pygopod): 1 : 1.5 – *A.p.* or 1 : 1.4 – *O.h.*.

Table 1 lists some differences in larval measurements (particular instars are not distinguished) between *A.talpa* and *O.haemorrhoa*.

## Discussion and summary

This paper gives a detailed description of the external structure of the hitherto unknown larval stage of *Amidobiatalpa* and *Oxypodahaemorrhoa* – Palearctic, myrmecophilous staphylinids belonging to the subfamily Aleocharinae – which are associated with the *Formicarufa* species group. It also gives the first description of the larva of *Amidobia*, and at present, the only complete, detailed account of the larval morphology of *Oxypoda*. The existing fragmentary descriptions of *Oxypoda* larvae, with only a few schematic drawings relating to just two species – *O.spectabilis* and *O.longipes* – were written 40–50 years ago (Pototskaya 1967, Topp 1978); in the context of contemporary comparative studies they are therefore practically useless. The diagnostic features presented above – 13 for *Amidobia* and 10 for *Oxypoda* – are based mainly on this description of the late larval instars of *A.talpa* and *O.haemorrhoa*. They mainly involve the structural details of the mouthparts, and in the case of the former taxon, of the antennae and spiracles as well. These features were established with reference to well-known larvae of other aleocharine species of diverse ecological preferences (Ashe and Watrous 1984, Staniec et al. 2009, 2010, 2017, 2018, Zagaja et al. 2014), among which anthill symbionts, represented by the two titular staphylinids, are deserving of particular scrutiny.

Relationships between myrmecophiles and their hosts exhibit varying degrees of advancement (Wasmann 1894, Hölldobler 1971, Hölldobler and Wilson 1990, Stoeffler et al. 2011, Staniec et al. 2017, Zagaja et al. 2017), which may be correlated with certain morphological adaptations in actively living developmental forms, including larvae. The data listed in Table 2 summarize the current state of our studies, which focus on the external structure of the larval stages of European myrmecophilous aleocharine species, especially in relation to the differing degrees of integration with their hosts (Stoeffler et al. 2011, Staniec et al. 2009, 2017, 2018, Zagaja et al. 2014). Five of the six species (genera) are known ant symbionts, associated with two species of these hymenopterans. Only *Dinaraeaaequata* Erichson W.F. 1837 is a typical saproxylic (non-myrmecophilous) beetle, totally unconnected with these social insects. In this configuration, therefore, it functions as a control species.

**Table 2.** Similarities and differences in external morphology between known late larval instars of some myrmecophilous and non-myrmecophilous Aleocharinae species. Abbreviations: A–article; a–anterior; arrg.–arrangement; As–apical seta of urogomphus; *ang.*–*angulata*; ap.–apical; c–central; cent. reg.–central region; cut. proc.–cuticular processes; Fe–femur; *haem.*–*haemorrhoa*; L–left mandible; l–long; Lr–length ratio; LWr–length to width ratio; Ma–mala; mod.–moderately; mic.–micro; mod.–moderately; Msn–mesonotum; Ns–number of setae; p–posterior; Pm–maxillary palp; Pnt–pronotum; preap.–preapical; poor.–poorly; *pub.*–*pubicollis* R–right mandible; S–segment; s–short; Sa–sensory appendage of antennal article II; Sas–subapical setae of urogomphus; St.–sternite; strong.–strongly; Tb–tibia; Ts–tarsungulus; Ug–urogomphus; w.–without; weak.–weakly; Wr–width ratio; ?–lack of data; *–body measurements of all known larval instars. Data based on Zagaja et al. (2014, 2017), Staniec et al. (2009, 2017, 2018), and the present study.

Ecological preferences	Myrmecophilous species					Non-myrmecophilous species
Host	* Lasius fuliginosus *	Formicarufa group				–
Tribe of Aleocharinae	Lomechusini		Oxypodini		Athetini	
Species	* Pella laticollis *	* Lomechusa pub. *	* Thiasophila ang. *	* Oxypoda haem. *	* Amidobia talpa *	* Dinaraea aequata *
level of integration with host	preadaptation to integration	peak of integration (symphile)	preadaptation to integration	non-integrated? (synoics)		–
**Character**						
Body length	4.30–4.80	4.99–6.70	2.72–4.40	2.46–3.32*	1.00–2.55*	3.01–3.78
Body shape	moderately elongate	dumpy	elongate	elongate	elongate	elongate
Cuticle	mod. sclerotized	membranous	mod. sclerotized	poor. sclerotized	poor. sclerotized	mod. sclerotized
Setae: structure	setose	blunt, jagged distally	setose	setose	setose	setose
**Head**						
Width	0.57–0.63	0.87–0.97	0.41–0.48	0.22–0.27*	0.16–0.23*	0.42–0.45
LWr	1:1	1:1.4	1:1	1.1:1	1:1.2	1:1
Ocelli	present	absent	present	present	present	present
Ns: dorsal side	40	70	42	40	40	40
Sides	distinctly rounded	distinctly rounded	distinctly rounded	weakly rounded	distinctly rounded	distinctly rounded
Wr of head and Pnt	1:1.3	1:1.5	1:1.3	1:1.1	1.1:1	1:1.1
**Antenna**						
Lr of AI-III	1.4:2.3:1	1.2:1.7:1	1.5:1.9:1	1.3 : 1.6 : 1	1.3:1.9:1	1:1.7:1
LWr of AI, AII, AIII	1.2:1/2.4:1/2.2:1	1:2.8/1:1.1/1:1.6	1.2:1/1.4:1/2:1	1:1.2/1.5:1/1.6:1	1:1/1.5:1/1.5:1	1:1/1.8:1/1.4:1
LWr of Sa	1.5:1	1.4:1	2.1:1	1.9:1	1.6:1	1.8:1
Lr Sa and AIII	1:1.6	1.1:1	1:1.1	1:1.3	1.1:1	1:1
**Labrum**						
Shape	trapeziform	trapeziform	semi-circular	semi-circular	rectangular	trapeziform
Anterior margin	almost straight	slightly rounded	distinctly rounded	distinctly rounded	excised in the centre	cent. reg. protruding, crenate
Seta Ld2: structure	mic. setose	macro, setose	macro, peg shaped	mic. verrucous	mic. spiniform	mic. spiniform
Labium and clypeus	separated	fused	separated	separated	separated	separated
**Epipharynx**						
Cuticular processes: No.	well above 200	well above 200	150–200	100–150	about 20	50–80
Cut. proc.: length	short	short	short	short	long	short
Cut. proc. in central area	absent	present	absent	absent	absent	present
No. of pores of a/c/p	2/8 (in 1 row)/ 4	?	1–2/9/4	2/6 (in 2 rows)/2	0/2/0	2/4/4
**Mandible**						
Shape	slender, bent weakly	slender, strong, bent	slender, weak, bent	slender, strong, bent	stocky, mod., bent	slender, weak, bent
Interior edge ap. tooth	slightly undulating	smooth	smooth	serrate	smooth	smooth
Edge below preap. tooth	slightly undulating	smooth	smooth	serrate	smooth	serrate
No. of preap. teeth	1-R and 1-L	0	1-R and1-L	R-1 and L-1	R-2 and L-2	4-R, 5-L
Number/length of setae	2/almost equal	3/almost equal	2/almost equal	2/different	2/almost equal	2/equal
**Maxilla**						
Mala: ant. marg. with:	27–31 equal teeth	8 equal setae	20 teeth (11 small)	11 teeth (1 big)	12 teeth (4 small)	23 teeth (15 small)
Mala: shape	wide, equilateral, sclerotized	lobar, membranous	slender, slightly dilated anteriorly	slender, slightly narrowed anteriorly	wide, distinctly dilated anteriorly	slender, distinctly dilated anteriorly
Ma: No. cut. proc./arrg.	about 80/singly	numerous/in rows	55–60/singly	15/singly	25/ singly	40/singly
Stipes and mala	fused	separated	separated	fused	fused	separated
Pm: Lr A I-III	1.7:1:2.3	1.4:1:2.0	1.6:1:2	1.5:1.0:2.0	1.1:1.0:2.0	1.6:1:2.2
Pm: LWr of AI-III	2.2:1/1.4:1/5.2:1	1:1.8/1:1.9/2.4:1	1.5:1/1.3:1/4.7:1	1.4:1/1.3:1/3.8:1	1.3:1/1.5:1/5.2:1	1.8:1/1.5:1/6.8:1
Lr: Pm and Ma	1.5:1	1:1.2	1.1:1	1.1:1	1.2:1	1.2:1
**Labium**						
Lg: shape	transverse, short,	transverse, very short	finger-like, 1.8 × as long as wide	finger-like, 1.9 × as long as wide	domelike, as long as wide	finger-like, 2.5 × as long as wide
Lg/anterior margin	sinuate	rounded	truncated	truncated	truncated	truncated
Lr: Lg and Lp	1:2.2	1:3.9	1:1.9	1:2	1:1.5	1:1
Lg and Pmnt	fused	fused	separated	fused	separated	separated
Pl: Lr A I and II	1:1.3	1:1	1:1.6	1:2.1	1:2.4	1:2.1
Pl: LWr of AI/AII	1.1:1/2.4:1	1:1/1.6:1	2:1/3.2:1	1:1/3.1:1	1:1.4/2.9:1	1.1:1/3.5:1
**Thorax**						
Ns: Pnt, Msn	52, 40	110, 80	52, 38	50. 36	50, 36	50, 38
Lr: Fe, Tb, Ts	2.3:2.2:1	2.2:1.9:1	2.1:2.2:1	2.4:2.0:1.0	1.8:1.7:1.0	1.9:2.2:1
LWr: Fe, Tb, Ts	4.4:1/4.5:1/5.2:1	2.2:1/2.3:1/3.0:1	3.6:1/5.2:1/5.2:1	2.9:1/ 3.0:1/3.5:1	2..6:1/4.3:1/5.6:1	3.0:1/5.3:1/7.1:1
Ns: Fe, Tb, Ts	8, 9, 2	30–34, 22–25, 2	8, 9, 2	8, 9, 2	8, 9, 2	7, 9,2
**Abdomen**						
Ns: Tergite I, II-VII	30, 30 each	70, 80 each	30, 32 each	28, 32	28, 32	32 each
Ns: St. I, each II-VIII	16, 20	100, 110	14, 20 each	16, 20	16, 20	16, 20 each
Urogomphi	present	absent	present	present	present	present
Ug: Lr AI, AII, Sap	1:1.1:1.2	–	1:2.2:2.6	2.4:1.0:4.8	1.4:1.0:3.1	1:1.5:1.7
LWr AII	2.7:1	–	3.7:1	2.5:1	2.8:1	4.1:1
Lr Ug (w. As) to S X	1:1.7	–	1:1	1:1.4	1:1.5	1:1.5

By far the largest number of characteristic features of the external structure, compared with other myrmecophilous and non-myrmecophilous aleocharines, were found in the larva of the symphilous genus *Lomechusa* Brisout de Barneville Ch.N.F., 1860 (Table 2). These beetles are the most highly integrated with their hosts, both behaviourally and morphologically (Hölldobler 1967, 1970, Parmentier et al. 2014, Parker 2016), and this also applies to the larval stages (Staniec et al. 2017). The morphological adaptations of their larvae are associated with the specific conditions prevailing within the anthill: absence of ocelli, a white body, and the close and continuous interaction in the host-guest system (e.g. absence of urogomphi; dense, asymmetrical chaetotaxy; membranous cuticle; short legs; some elements of mouthparts shortened). In addition, this distinctive structure of the larval stage of *Lomechusa* is accompanied by a passive lifestyle, possible trophallaxis and chemical mimicry.

The classification of the degree of integration of the other four myrmecophilous species of Aleocharinae is not so obvious. Nonetheless, there do seem to be certain differences between them in this respect. Stoeffler et al. (2011), referring to Hölldobler (1981), suggests that in *Pellalaticollis* there is behavioural pre-adaptation towards a closer relationship with the host. Evidence for this could be the presence in adult beetles, in contrast to the other representatives of this genus, of glands modifying the behaviour of the ants, which enables the beetles to live unmolested in the near neighbourhood of the anthill. Again, on the basis of existing classifications (Wasmann 1894, Kistner 1979) and observations of different behavioural aspects of *Thisophilaangulata*, Zagaja et al. (2017) place this species among host-integrated myrmecophiles. However, Stoeffler et al. (2011) demonstrated that the relations of *T.angulata* with ants resemble a pre-adaptation (initial phase) to a closer relationship with them, rather than complete integration, as in the case of *P.laticollis*. The relationships with hosts of the smallest of this group of beetles – *Amidobiatalpa* and *Oxypodahaemorrhoa* – were not examined. It seems, however, that according to Wasmann’s (1894) classification, they are probably synoics, that is, myrmecophiles feeding on detritus or other organisms inhabiting the anthill. Opportunistically, they may also consume eggs and small larvae of ants. Because they are small and highly mobile, these beetles are probably completely ignored by the host (Parmentier et al. 2015, own observations).

In view of the above it cannot be surprising that, with the exception of *Lomechusa*, discussed earlier, the other myrmecophilous larvae analysed here do not possess any outstanding features distinguishing them from non-myrmecophilous species (Table 2). Therefore, the morphological differences between the aleocharine larvae examined here are probably a reflection of the biotic and abiotic conditions specific to the particular microhabitat they occupy rather than of more general habitat preferences (myrmecophily or non-myrmecophily). The crucial aspect of this situation thus appears to be the trophic specialization of these tiny predators, a question as yet incompletely understood. That is why the greatest number of differences between them concerns the structural details of the mouthparts (e.g. shape of labrum and ligula, structure of epipharynx, mandibles, mala, length of articles of maxillary and labial palps), this differentiation being strictly linked with the food resources these larvae consume (Table 2). Other characteristic features of these larvae include the detailed structure of the antennae, the urogomphi, less often the structure of the spiracles (*A.talpa*) and head shape (*O.haemorrhoa*). The present morphological analysis has not revealed any features characteristic of the several tribes (Table 2). This might indicate, on the one hand, the need to reassess the systematics of the higher taxonomic units in Aleocharinae, but on the other, that the larval characteristics of these rove beetles are of minimal usefulness in phylogenetic studies.

Therefore, as studies to date have shown, the characteristic morphology of the aleocharine larvae examined to date is not due to their myrmecophily alone. Likewise, the larval stages of myrmecophiles, which exhibit behavioural pre-adaptations to integration with host ants (*P.laticollis*, *T.angulata*), do not possess any visible external structural features pointing to associations with ants (Staniec et al. 2009, Zagaja et al. 2014). By contrast, symphiles, i.e. aleocharine species wholly integrated with their hosts and obligatorily dependent on them (e.g. *Lomechusa*), do exhibit a far-reaching restructuring of the body, particularly that of the larva. The unique morphological features of larvae (Table 2) are the result of advanced adaptations to life in an anthill and to constant interactions with their inhabitants (Hölldobler 1967, Parker 2016, Staniec 2017). In this context, the structure of the newly-described larvae of *Amidobia* and *Oxypoda* is typical of tiny, predacious Aleocharinae, not associated with ants (Table 2). In all probability, because they are highly mobile and very small (max. lengths up to 2.6 and 3.3 mm respectively), they are, like the adult forms, entirely ignored by the worker ants. They can thus live unmolested among ants without the need to possess the morphological adaptations that have evolved in the larger and slower symphiles. Similar, co-existential strategies in other small insect species associated with ants were described by Parker (2016) and Parmentier et al. (2015).

This analysis of the comparative morphology of known myrmecophilous aleocharine larvae in the context of the type of interaction with hosts is merely a preamble to far more extensive research on this subject. Unfortunately, as knowledge of the larval stage, not only of myrmecophilous but of other members of this very numerous staphylinid subfamily, remains fragmentary, the formulation of more comprehensive generalizations is as yet not possible. Moreover, there is still no information whatsoever on the detailed external larval structure of a number of other interesting, symbiotic European aleocharines. This situation can be illustrated by the genus *Dinarda* Leach W.E., 1819. Its members exhibit behaviour testifying to quite an advanced degree of integration with hosts, including the possibility of their being fed by ants on the principle of regurgitation (Hölldobler and Wilson 1990). It may well be that the mouthparts of *Dinarda* larvae are adapted to this form of feeding in the same way as in *Lomechusa* (Staniec et al. 2017) and that they possess other features emerging from their close relationships with ants. Where the degree of advancement of integration with hosts is concerned, such features would help to place *Dinarda* right after members of *Lomechusa*, and certainly in front of the other myrmecophiles listed in Table 2. But further studies are needed in order to find a definitive answer to this question.
